# Yield Difference between Different Cultivation Techniques under Ultrasonic Treatment Driven by Radiation Use Efficiency

**DOI:** 10.3390/plants13172510

**Published:** 2024-09-06

**Authors:** Sicheng Deng, Qichang Gu, Yizhu Wu, Wentao Yi, Jian Lu, Ligong Peng, Xiangru Tang

**Affiliations:** 1 State Key Laboratory for Conservation and Utilization of Subtropical Agricultural Bioresources, South China Agricultural University, Guangzhou 510642, China; dengsc@stu.scau.edu.cn (S.D.); guqichang@stu.scau.edu.cn (Q.G.); wyzhu@stu.scau.edu.cn (Y.W.); ywt1999@stu.scau.edu.cn (W.Y.); lj15090709256@126.com (J.L.); plg@stu.scau.edu.cn (L.P.); 2 Scientific Observing and Experimental Station of Crop Cultivation in South China, Ministry of Agriculture and Rural Affairs, Guangzhou 510642, China; 3 Guangzhou Key Laboratory for Science and Technology of Fragrant Rice, Guangzhou 510642, China

**Keywords:** ultrasonic, cultivation technique, radiation use efficiency, grain yield

## Abstract

Ultrasonic treatment and optimal cultivation techniques are both conducive to the high yield of super rice in South China. Many previous studies have shown that the increase in intercepted photosynthetically active radiation (IPAR) and radiation use efficiency (RUE) is an important reason for high rice yield. Field experiments were conducted over two years to evaluate the effects of IPAR and RUE on the yield under different treatments (CK: conventional cultivation technique without ultrasonic treatment; T1: conventional cultivation technique with ultrasonic treatment; T2: super rice-specific cultivation technique without ultrasonic treatment and T3: super rice-specific cultivation technique with ultrasonic treatment), with two representative rice varieties, Wufengyou-615 (WFY) and Jingnongsimiao (JNSM) during the late seasons of rice cultivation in South China. The super rice-specific cultivation technique and the ultrasonic treatment could significantly increase the yield, which was significantly (*p* < 0.01) and positively correlated with panicle number, grain-filling rate, and aboveground total dry weight. The higher grain yield depended more highly on higher RUE in the mid-tillering stage and maturity stage. The results of multiple-regression models also showed that the contributions of IPAR and RUE to yield were significant (*p* < 0.01). Conclusively, IPAR and RUE contributed a lot to yield progress of super rice in both super rice-specific cultivation techniques with fewer times of topdressing and ultrasonic treatment in South China. It is worth further studying how to reasonably improve the RUE of high-RUE varieties through other means.

## 1. Introduction

Rice (*Oryza sativa* L.) is one of the most important food crops. China is the largest consumer of rice while 50% of the world’s population also relies on it as the staple food [1,2]. Super rice breeding was proposed by the Ministry of Agriculture of China in 1996, which mainly emphasized the construction of ideal plant types and the utilization of strong heterosis between indica and japonica subspecies [3]. Super rice refers to new rice breeds that possess high yield potential, significantly outperforming existing rice varieties under ultra-high-yield cultivation and management conditions, and are also characterized by multiple resistances and superior quality [4]. Although there is no genetic and botanical difference between super rice varieties and inbred rice varieties, the high yield potential of super rice is more prominent with better resistance and higher quality characteristics. Compared with inbred rice cultivars, the yield of super rice can be greatly improved under super high-yield cultivation and management conditions [5,6,7]. 

In the long history of cultivation, the experience and technology of rice production have been accumulated, and a series of cultivation techniques suitable for high yield of rice have been formed in various places, such as rice intensive cultivation technique [8], “San-ding” cultivation technique [9] and “three controls” fertilization technology [10], etc. In South China, a series of characteristic high-yield cultivation techniques represented by super rice-specific cultivation techniques have also been formed [11]. Furthermore, considering the soil fertility and the characteristics of rice fertilizer demand of each cultivation area, the use of rice-specific fertilizer is also an important way to increase yield [12]. Rice-specific fertilizer can effectively improve the physical and chemical properties of soil, promote the absorption capacity of rice fertilizer, and prolong the functional period of leaves [13]. Some trace elements and organic matter contained in the specific fertilizer also have a certain effect on the promotion of the morphology of rice leaves, SPAD (Soil and Plant Analyzer Development, which can directly reflect the relative content of chlorophyll in plant leaves) value in leaves, dry matter accumulation and lodging resistance [14,15]. Some cultivation techniques aim to enhance seedling vigor. For example, using a seedling strengthening agent can improve seedling quality and resistance, increase seedling survival rates, and shorten the post-transplanting seedling stage, therefore promoting seedling development and laying a solid foundation for high yields [15]. Spraying foliar fertilizer during the heading stage can prolong the leaf’s functional period, enhance source function, and improve sink activity, offering an efficient and convenient method to increase yield [16]. Therefore, selecting suitable cultivation techniques for different regions is an efficient way to boost yields.

Ultrasonic is a kind of mechanical wave with high frequency, short wavelength, high energy and strong penetrating power [17]. Ultrasonic seed treatment technology has been widely adopted globally, achieving significant results [18]. Ultrasonic seed treatment technology can break seed dormancy and enhance germination rate by physical approaches [19,20]. Under ultrasonic vibration, plant cell structure and function are altered, influencing cellular physical properties and physiological activities. This promotes oxidation, reduction, decomposition, and synthesis within plant cells, enhancing seed germination rates and ratios [21]. Physiologically, ultrasonic treatment improves seed vigor and stress resistance [22]. Huang et al. [23] declared that ultrasonic treatment can enhance rice leaf antioxidant activity and reduce peroxide content, therefore improving rice quality and resistance. Moreover, ultrasonic treatment can alter cell membrane permeability and promote seed expansion and starch hydrolysis, leading to increased effective panicles, spikelets per panicle, and, ultimately, higher yields [24].

Crop yield is influenced by biological and abiotic factors such as sunlight, climate, available water, and fertilizers. Solar radiation, a crucial indicator of sunlight energy, significantly impacts crop growth, development, and productivity. A total of 90~95% of the total dry matter of crops comes from the photosynthetic biofixation of CO_2_ [25]. Crop yield is generally calculated as the product of biomass production and harvest index (HI), while biomass production can be expressed by the product of photosynthetically active radiation (PAR) intercepted by crop canopy and radiation use efficiency (RUE) [26]. From a physiological point of view, and taking into account the potential for cultivation, the aboveground biomass production under non-stress conditions is closely related to intercepted photosynthetically active radiation (IPAR) and RUE [27,28]. Therefore, without considering the root system, the yield can also be considered to be the product of IPAR, RUE, and HI. RUE is the efficiency of converting IPAR into dry matter, which is an important index to understand crop growth and yield [29]. The potential of HI has been greatly explored in past studies, and it is very difficult to further improve it presently [30]. Many previous studies have shown that the increase in IPAR and RUE is an important reason for high rice yield [26,31,32]. Therefore, based on maintaining HI, further increasing RUE and IPAR is an effective way to increase yield.

To explain the yield difference of rice between different cultivation techniques under ultrasonic treatment by IPAR and RUE, we conducted a two-year field experiment on two representative super rice varieties in South China. The purposes of this study were to (1) compare the effects of ultrasonic treatment and different cultivation techniques on super rice yield, IPAR, and RUE and (2) determine the relationship between yield, aboveground total dry weight, IPAR, and RUE of super rice under different treatments at different growth stages.

## 2. Results

### 2.1. Yield and Yield Components

**Figure 1:**
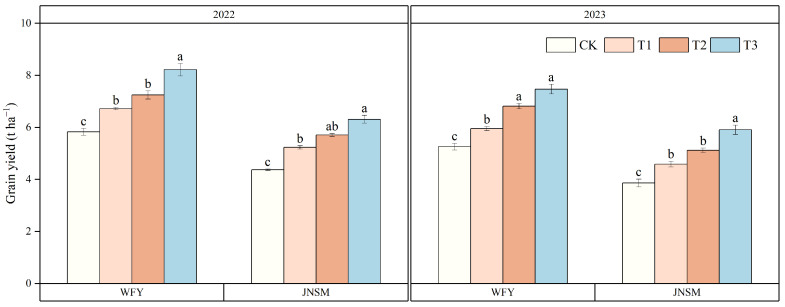
Grain yield of two rice varieties under different treatments in 2022 and 2023. Vertical bars indicate standard errors (n = 3). Different lowercase letters indicate statistical differences among treatments at *p* < 0.05.

The super rice-specific cultivation technique and ultrasonic treatment could significantly increase the yield of super rice in two years (Figure 1 and Table 1). The yield of the T3 treatment was the highest, followed by T2, T1, and CK. In WFY, the yield of the super rice-specific cultivation technique was 26% higher than that of the conventional cultivation technique, while the yield of ultrasonic treatment was 14% higher than that of non-ultrasonic treatment. In JNSM, the yield of the super rice-specific cultivation technique was 27% higher than that of the conventional cultivation technique, while the yield of ultrasonic treatment was 15% higher than that of non-ultrasonic treatment. The average yields in 2022 and 2023 were 6.19 t ha^−1^ and 5.26 t ha^−1^, respectively.

**Figure 2:**
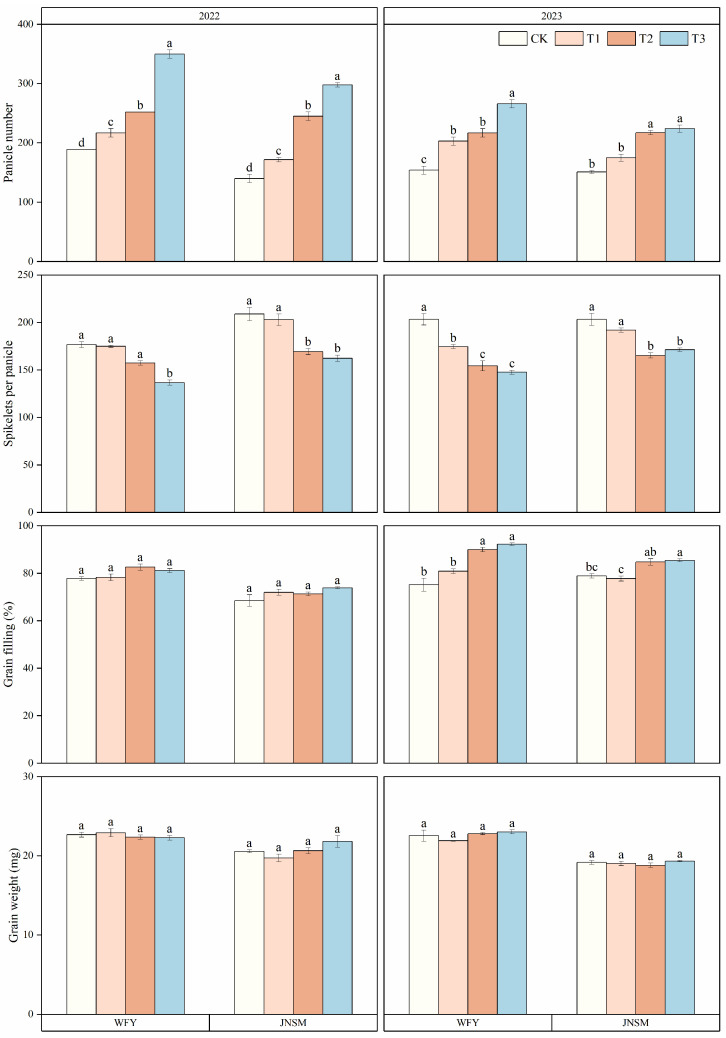
Yield components of two rice varieties under different treatments in 2022 and 2023. Vertical bars indicate standard errors (n = 3). Different lowercase letters indicate statistical differences among treatments at *p* < 0.05.

Panicle number, spikelets per panicle, and grain-filling rate were significantly affected by cultivation techniques and ultrasonic treatment (Figure 2 and Table 1). The super rice-specific cultivation technique and ultrasonic treatment could significantly increase the panicle number but had a significant negative effect on the increase of spikelets per panicle. The effect of ultrasonic treatment on grain-filling rate was negligible, but the super rice-specific cultivation technique could obviously improve it. Compared with the conventional cultivation technique, the super rice-specific cultivation technique increased the grain-filling rate by 4.2% in 2022 and 12.7% in 2023, respectively. It showed that the increase in yield was mainly caused by increasing the panicle number and grain-filling rate.

### 2.2. Aboveground Biomass, Growth Periods, and Crop Growth Rate 

**Figure 3:**
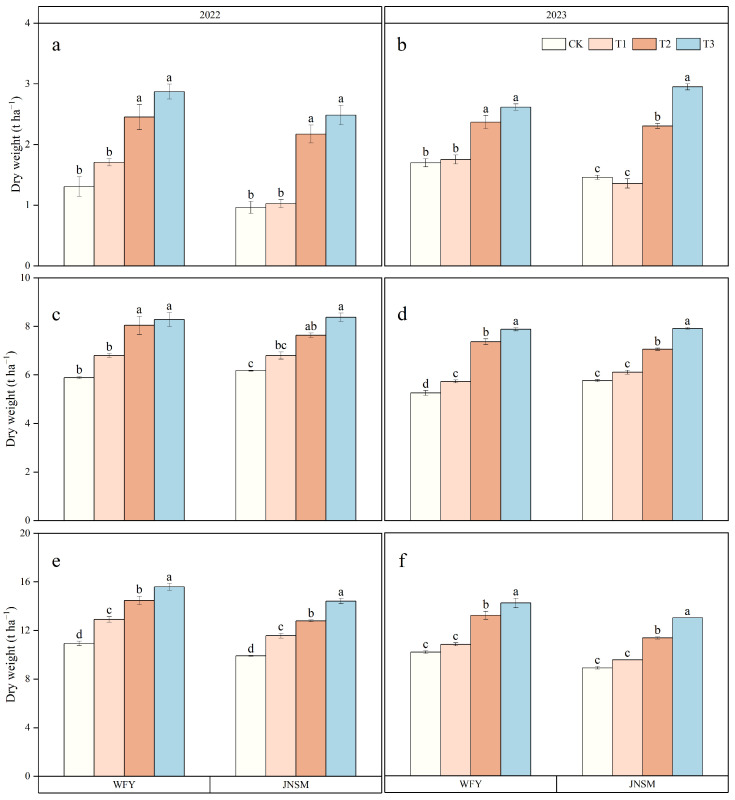
Aboveground total dry weight (TDW) of two rice varieties under treatments in MT (**a**), FL (**c**), and MA (**e**) in 2022, and MT (**b**), FL (**d**), and MA (**f**) in 2023. Vertical bars indicate standard errors (n = 3). Different lowercase letters indicate statistical differences among treatments at *p* < 0.05.

**Figure 4:**
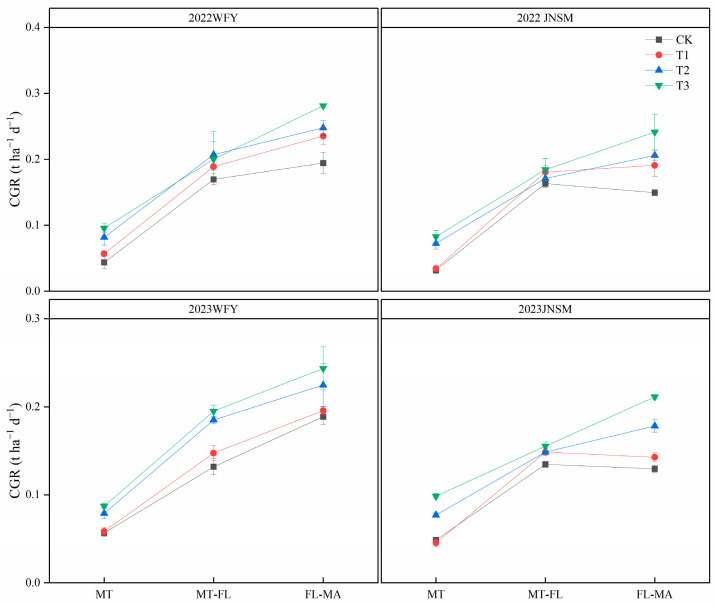
Crop growth rate (CGR) of two rice varieties under different treatments in 2022 and 2023. Vertical bars indicate standard errors (n = 3).

Aboveground total dry weight at MT, FL, and MA significantly (*p* < 0.05) differed with treatments (Figure 3). At all growth stages of both varieties in both years, T3 had the highest dry weight, followed by T2, T1, and CK in descending order. The cultivation techniques had the greatest effect on the dry weight at MT, and the dry weight under the super rice-specific cultivation technique increased by 79% compared with the conventional cultivation technique at MT. The effect of ultrasonic treatment on dry weight originated from MT, and the dry weight under ultrasonic treatment increased by 13.8% compared with that under non-ultrasonic treatment at MT. In addition, except for MT, aboveground total dry weight in 2022 was 9.2% and 14.6% higher than that in 2023 at FL and MA, respectively. Growth periods varied (*p* < 0.05) between different varieties (Table 2). The growth period of WFY was 101 d, and the growth period of JNSM was 97 d and 98 d in 2022 and 2023, respectively. All treatments had no significant effect on the growth periods. Crop growth rate (CGR) varied between different treatments in the different growth periods (Figure 4). Ultrasonic treatment mainly improved CGR in the period of FL–MA and the super rice-specific cultivation technique significantly increased CGR in the period of MT–FL and FL–MA. 

### 2.3. IPAR, RUE, and HI 

**Figure 5:**
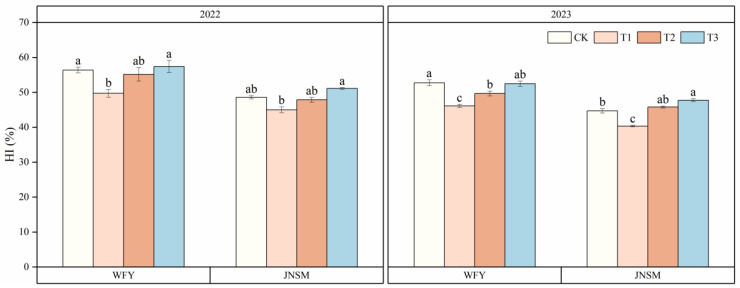
Harvest index (HI) of two rice varieties under different treatments in 2022 and 2023. Vertical bars indicate standard errors (n = 3). Different lowercase letters indicate statistical differences among treatments at *p* < 0.05.

Intercepted photosynthetically active radiation (IPAR) and radiation use efficiency (RUE) in different growth periods varied significantly (*p* < 0.05) between different treatments (Table 1 and Table 2). The results indicated that the two varieties under T3 treatment generally had the highest IPAR at each growth stage in two years, followed by T2, and the plants under CK were the lowest. The IPAR of the whole growth stage (WGS) of the plants under T3 treatment was 2.7%, 8.2%, and 11.5% higher than that under T2, T1, and CK, respectively. The RUE-WGS were the highest in T3, followed by T2, and the lowest in CK, and the differential trend of RUE in other periods was also the same, but differences between treatments in the MT–FL period were negligible. The RUE-WGS of T3 was 7.3%, 18.6%, and 28.4% higher than that of T2, T1, and CK, respectively. It also showed that the RUE in the FL–MA period was the highest, which was 12.2% and 49.5% higher than that in MT–FL and MT, respectively. In addition, it was found that although the IPAR-WGS in 2023 was 22.4% lower than that in 2022, the RUE-WGS in 2023 was 14.3% higher than that in 2022. Harvest index (HI) of two varieties also varied significantly (*p* < 0.05) between different treatments in two years (Table 1 and Figure 5). The HI of T3 and CK was the highest, and the difference was generally not obvious, followed by T2, and the HI of T1 was the lowest. The HI in 2022 was 9.3% higher than that in 2023.

### 2.4. Correlation Analyses of Grain Yield and Population Growth Parameters 

**Figure 6:**
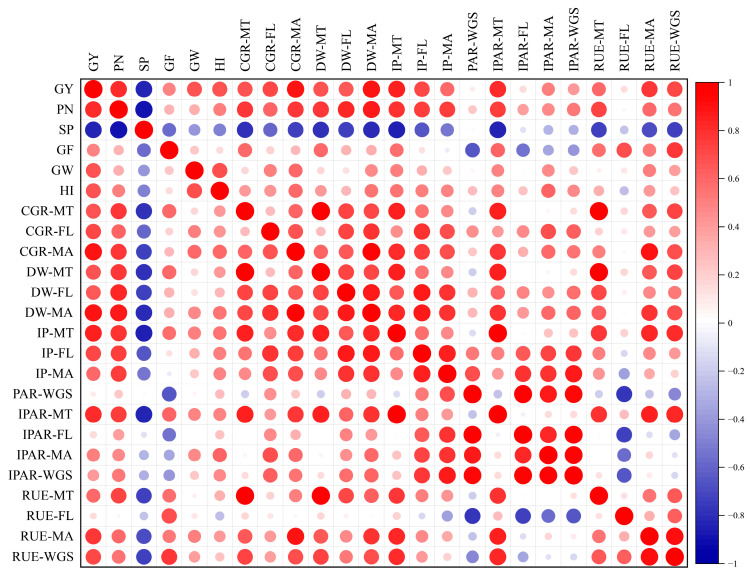
Correlation matrix of various grain yield and population growth parameters (n = 48). GY, grain yield; PN, panicle number; SP, spikelets per panicle; GF, grain filling; GW, 1000-grain weight; HI, harvest index; MT, mid-tillering stage; FL, flowering stage; MA, maturity; WGS, whole growth stage; CGR, crop growth rate; DW, dry weight; IP, light interception percentage; PAR, photosynthetically active radiation; IPAR, intercepted photosynthetically active radiation; RUE, radiation use efficiency.

Grain yield, grain yield components, population growth parameters, and other parameters about solar radiation were shown in the correlation matrices (Figure 6). GY was significantly (*p* < 0.01) and positively correlated with PN, GF, GW, HI, CGR, and IP. Additionally, IPAR-MT, IPAR-MA, RUE-MT, RUE-MA, and RUE-WGS also had significantly (*p* < 0.01) positive correlations with grain yield.

### 2.5. Relationships between Grain Yield and Intercepted Radiation or RUE at Different Stages

**Figure 7:**
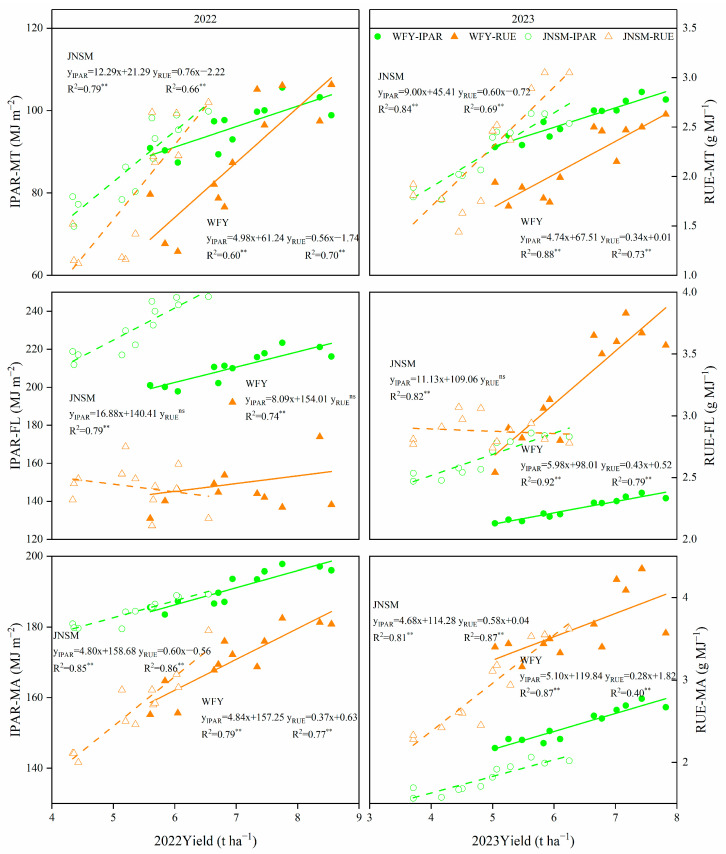
Relationships between grain yield and intercepted photosynthetically active radiation (IPAR) and radiation use efficiency (RUE) in MT, MT–FL, and FL–MA. Data were from all replicates across two years (n = 12). ** significant at *p* < 0.01; ns, non-significant.

Both IPAR and RUE were positively correlated with grain yield across different growth stages (MT, MT–FL, FL–MA), but these positive correlations also varied between different growth stages (Figure 7). The higher the slope of the fitted line in the figure, the lower the correlation. The results showed that the higher grain yield of WFY in 2022 was more closely related to RUE-MT (R^2^ = 0.70), RUE-MA (R^2^ = 0.77), IPAR-MT (R^2^ = 0.60), and IPAR-MA (R^2^ = 0.79), while the higher grain yield of WFY in 2023 also depended more highly on RUE-MT (R^2^ = 0.73), RUE-MA (R^2^ = 0.40), IPAR-MT (R^2^ = 0.88) and IPAR-MA (R^2^ = 0.87). The higher grain yield of JNSM in 2022 was more closely related to RUE-MT (R^2^ = 0.66), RUE-MA (R^2^ = 0.86), IPAR-MT (R^2^ = 0.79) and IPAR-MA (R^2^ = 0.85), while the higher grain yield of JNSM in 2023 also depended more highly on RUE-MT (R^2^ = 0.69), IPAR-MT (R^2^ = 0.84) and IPAR-MA (R^2^ = 0.81). In addition, compared with the period of MT, IPAR, and RUE, the period of FL–MA generally showed a greater effect on yield.

### 2.6. Evaluation of the Effects of IPAR, RUE, and HI on Grain Yield of Each Variety 

The following multiple-regression models were devised to quantify the relative contributions of IPAR, RUE, and HI to the grain yield of the two varieties, respectively (Table 3).

ln(Grain yieldWFY) = −4.669 + 0.798 ln(IPAR) + 0.793 ln(RUE) + 0.063 ln(HI)

ln(Grain yieldJNSM) = −4.379 + 0.985 ln(IPAR) + 0.811 ln(RUE) − 0.234 ln(HI)

ln(Grain yieldMean) = −5.747 + 0.439 ln(IPAR) + 0.695 ln(RUE) + 0.327 ln(HI)

The grain yield of both WFY and JNSM was significantly (*p* < 0.01) affected by IPAR and RUE. The regression coefficients of WFY were 0.798 and 0.793 to IPAR and RUE, indicating that higher values of both IPAR and RUE resulted in higher grain yields, while IPAR might have made a higher relative contribution to grain yield in WFY. The regression coefficients of JNSM were 0.985 and 0.811 to IPAR and RUE, likewise indicating that higher values of both IPAR and RUE resulted in higher grain yields, while IPAR might have made a higher relative contribution to grain yield in JNSM. The multiple-regression model of the mean indicated that increasing RUE was more likely to improve yield generally.

## 3. Discussion

Super rice is a kind of rice type with extremely high yield potential bred in China. How to release its high yield potential and study the reasons for its high yield has always been the focus of research [33]. The super rice-specific cultivation technique was developed by Professor Tang Xiangru’s team, which is conducive to the high yield of super rice in South China. This technique mainly maximizes the yield potential of super rice using seedling strengthening agents during the seedling stage, applying super rice-specific fertilizer after transplantation, and using foliar fertilizer during the heading stage [34]. As a means of physical regulation, ultrasonic treatment of rice seeds before seed soaking is also conducive to improving seed vigor, seedling rate, and seedling ratio, which is beneficial for ensuring the number of high-quality seedlings before transplanting and increasing yield finally [35]. This experiment is an attempt to try to further release the yield potential of super rice in South China using these two methods and explain the reasons. This study showed that both the super rice-specific cultivation technique and the ultrasonic treatment mainly increased the yield by increasing the number of panicles and grain-filling rate (Figure 2). In the super rice-specific cultivation technique, the application of fertilizer was concentrated on base fertilizer and tillering fertilizer, so more tillers were produced in the tillering stage compared with the conventional cultivation technique. Due to the use of seedling strengthening agents in the seedling stage, the number of high-quality seedlings was large. At the same time, the super rice-specific fertilizer could promote the early emergence of large tillers and the rapid growth of leaf area index, and spraying foliar fertilizer at the heading stage could also postpone the decrease of chlorophyll content in leaves at grain filling and maturity stage, strengthen the function of the leaf as “source” and the activity of grain as “sink”, thus increasing the number of effective panicles and the yield finally [36]. According to the study of Huang et al. [35], ultrasonic treatment could improve seed vigor and seedling quality and promote rice tillering after transplanting. Under T1 treatment, more effective panicles can also be formed because of the reasonable nitrogen management, but the spikelets per panicle decrease [37].

Previous studies have shown that the RUE of non-stressed rice canopy is one of the most promising traits in climatic factors to further increase yield, and the RUE of super rice is generally higher than that of inbred rice [32]. In this study, the increase of yield under optimized cultivation technique and ultrasonic treatment was accompanied by the corresponding increase of IPAR and RUE. The yield of the two super rice varieties was positively correlated with RUE and IPAR, which was consistent with previous studies [38,39]. These results showed that both the ultrasonic treatment and the super rice-specific cultivation technique increase the yield by increasing IPAR and further improving RUE. Because the growth period of each variety under different treatments in each year was the same, the PAR value from the solar was the same, and the increase of IPAR value in this experiment was mainly achieved by increasing the light interception percentage (IP). The realization of high IP was mainly due to the increase of effective panicle number (Figure 2), which is consistent with the results that ultrasonic and super rice-specific cultivation techniques could increase the effective panicle number. Although ultrasonic treatment could also improve RUE, the super rice-specific cultivation technique had a more significant effect on RUE (Table 2). Compared with the conventional cultivation technique, plants under the super rice-specific cultivation technique had higher IPAR and more radiation energy to utilize, while spraying foliar fertilizer at the heading stage could improve the grain-filling rate of rice, thus leading to higher RUE [40]. By analyzing the correlation between IPAR or RUE and yield at each growth stage, it was found that the correlation was stronger at the periods of MT and FL–MA, indicating that increasing IPAR and RUE after the tillering and heading stages was more conducive to improving the yield of super rice (Figure 7). The improvement of IPAR by ultrasonic treatment originated from the tillering stage, and the super rice-specific cultivation technique also increased IPAR by increasing effective panicles at the tillering stage and increased RUE after heading by spraying foliar fertilizer at the heading stage, which showed that in the perspective of radiation utilization, both two treatments were highly effective methods to increase yield.

Increasing biomass production and harvest index (HI) play an important role in effectively increasing crop yield [25]. High aboveground total dry weight and high HI are the basis and conditions for high yield, respectively. It is very important to coordinate the relationship between dry weight and HI for improving rice yield [41]. The difference in dry weight between samples can be attributed to different IPAR and RUE or the combination of these two factors [42]. In this experiment, aboveground total dry weight (TDW) was also positively correlated with IPAR and RUE, which was consistent with previous studies. HI was also positively correlated with yield, IPAR, and RUE in general (Figure 6), but it was the highest under T3 and CK treatments and the lowest under T1 treatment (Figure 5). The reason HI under CK treatment was higher than that under T1 and T2 treatment might be that the panicle number under CK was the least, and the fertilizer applied in later stages was more concentrated in the reproductive growth, causing the development of panicles to be better than that of vegetative organs. Under T2 and T3 treatments, not only spraying foliar fertilizer at the heading stage was beneficial to grain filling, but sufficient IPAR ensured enough development of each panicle, so the HI of plants under T2 and T3 treatments was also higher. The contribution of HI, IPAR, and RUE to yield was evaluated by constructing multiple-regression models of different varieties (Table 3). It was found that it was more realistic to increase yield by increasing IPAR and RUE than by increasing HI in both varieties [30,32]. Previous studies have also pointed out that RUE is a relatively simple method to simulate the net increase of crop dry matter, with a typical value of 2.2 g MJ^−1^ in inbred rice, while HI, which is also related to yield increase, has a value of only 0.32 [43,44]. Our experiments found that the HI and the RUE values of super rice were both significantly higher than those of inbred rice, which revealed that one of the reasons for the high yield of super rice might be its higher RUE and HI values. These findings clearly indicated that, compared to enhancing HI, it was a more reasonable approach to increase yield by improving the IPAR and RUE of super rice through the application of ultrasonic treatment and super rice-specific cultivation technique.

This study also encountered some limitations. Solar radiation is affected by latitude, weather conditions, altitude, and sunshine time. In this experiment, although the yield, HI, and PAR in 2023 were lower than those in 2022, the overall RUE was higher in 2023. It is speculated that the development of panicles was seriously hindered due to severe weather conditions in the late season of 2023, but the decrease of TDW was alleviated due to the guarantee of sufficient fertilizer in the soil. Additionally, there are still few studies on RUE and yield of double cropping rice in South China. Therefore, it is necessary to further explore the effects of sunshine intensity, temperature regulation and soil nitrogen supply on the contribution of RUE and IPAR to biomass production and yield in South China.

## 4. Materials and Methods

### 4.1. Field Experimental Details

Two rice cultivars, Wufengyou-615 (WFY, indica, 2012, Guangzhou, China) and Jinnongsimiao (JNSM, indica, 2010, Guangzhou, China), were used in the experiment. These cultivars, widely planted in South China, are both defined as super rice and have similar growth periods.

Field experiments were conducted in the late season of 2022 and 2023 at the Experimental Research Farm in Zengcheng, College of Agriculture, South China Agricultural University, Guangzhou, China (113°64′ E, 23°24′ N, altitude 11 m). The nutrient composition of the soil in the experiment field, which was sandy loam, was as follows: 1002 mg kg^−1^ total N, 989 mg kg^−1^ total P, 19,563 mg kg^−1^ total K, 62.4 mg kg^−1^ available P, 93 mg kg^−1^ available K, and 18,263 mg kg^−1^ organic C.

Treatments were arranged in a split-plot design, with treatments as the main plots and cultivars as the subplots. The experiment was performed with 3 duplications, and the size of the subplot was 120 m^2^. Pre-germinated seeds were sown in pot seedling trays at 25 g m^−2^, and seedlings with three leaves were transplanted by a pot seedling transplanter at 18 days old to field plots with 16 × 30 cm hill spacings and two seedlings per hill. This study involved four treatments: conventional cultivation technique without ultrasonic treatment (CK), conventional cultivation technique with ultrasonic treatment (T1), super rice-specific cultivation technique without ultrasonic treatment (T2), and super rice-specific cultivation technique with ultrasonic treatment (T3). The ultrasonic treatment was that seeds were pre-treated by an Ultrasonic Crop Production Tunnel Processor for 5 min with 20–40 kHz mixed frequency (5ZCG-50, Guangzhou Golden Rice Agricultural Science & Technology Co., Ltd., Guangzhou, China). The time for ultrasonic treatment of seeds was two days before sowing. The conventional cultivation technique was the locally popular cultivation technique. Super rice-specific cultivation technique was developed by Professor Tang Xiangru’s team at South China Agricultural University through years of research. It is an integrated cultivation technique specifically for super rice, with the application of seedling strengthening agents, super rice-specific fertilizer, and foliar fertilizer. The comparison of two cultivation techniques is shown in Table 4. A 30 cm high and wide bund was built between different cultivation techniques. Field management followed standard cultural practices. Insect injuries and diseases were intensively controlled with chemicals to avoid biomass and yield losses.

**Figure 8:**
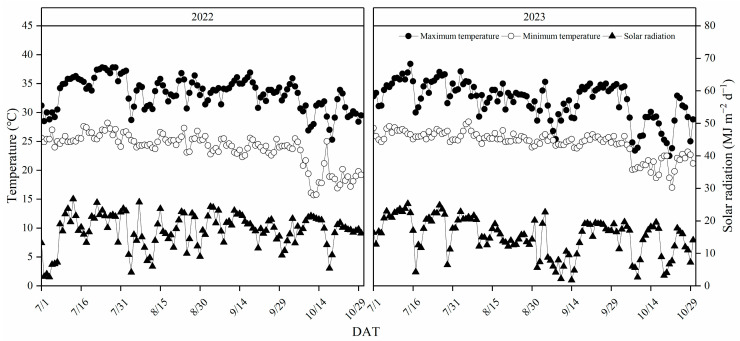
Maximum temperature, minimum temperature, and solar radiation from July to October in 2022 and 2023 in Zengcheng, Guangdong Province, China. DAT, growth date.

Partial meteorological data from July to October 2022 and 2023 were also statistically analyzed (Figure 8). The mean values of maximum temperature, minimum temperature, and daily solar radiation were 33.3 °C, 23.8 °C and 17 MJ m^−2^ in 2022 and 32.1 °C, 24.7 °C and 15 MJ m^−2^ in 2023, respectively. The total solar radiation was 2080 MJ m^−2^ and 1861 MJ m^−2^ in 2022 and 2023, respectively. There was negligible difference in daily average temperature and daily solar radiation between the two years, but the total solar radiation in 2022 was significantly higher than that in 2023, and the total solar radiation during the growth period in 2022 was 20% higher than that in 2023.

### 4.2. Measurement of Aboveground Biomass Production, Grain Yield, Yield Components and Crop Growth Rate

Samples were taken at the mid-tillering stage (MT), booting stage (BT), flowering stage (FL), and maturity (MA) from eight randomly selected hills in each subplot to determine aboveground dry weight. At the maturity stage, eight plants were sampled diagonally in each subplot to measure HI and yield components. The panicle number of each plant was counted, and the average number was calculated to determine the final panicle number (m^2^). Then, plants were divided into straw and panicles. The panicles were threshed and separated into filled spikelets and unfilled spikelets manually. The number of spikelets was measured by Plant Seed Analyzer (OPTO-Agri, Optomachines, Riom, France). The dry weight of the straw, rachis, and the filled and unfilled spikelets were measured after oven-drying at 80 °C to constant weight. Spikelets per panicle (spikelet number/panicle number), grain-filling percentage (filled spikelet number/total spikelet number × 100%), and HI (filled spikelet dry weight/aboveground total dry weight × 100%) were also calculated. The grain yield of each treatment was measured by harvesting rice from a 5 m^2^ area with three duplications and adjusted according to the moisture content of 14%. The grain moisture content was measured by Grain Moisture Meter (PM-8188 A, Kett, Tokyo, Japan). Crop growth rate (CGR) was calculated by following formula [45]:

CGR (g m^−2^ d^−1^) = (DW_2_ − DW_1_)/(t_2_ − t_1_)

In the formula, DW_1_ and DW_2_ are the aboveground biomass (g m^−2^) measured at times t_1_ and t_2_, respectively, while t_1_ and t_2_ refer to the specific dates of sampling.

### 4.3. Measurement of Intercepted Radiation and Radiation Use Efficiency

Canopy light interception was measured between 11:00 and 13:00 at MT, FL, and MA, using the SunScan Canopy Analysis System (Delta-T Devices Ltd., Burwell, Cambridge, UK). In each treatment, the incoming light intensity and the light intensity inside the canopy were measured by placing the light bar halfway slightly above the canopy and water surface between two rows, respectively. This measurement was duplicated six times. Canopy light interception was defined as the value of [(Incoming light intensity-Light intensity inside canopy)/Incoming light intensity × 100%]. The IPAR during each growth stage was defined as the value of [1/2 × (Canopy light interception at the beginning of the growth stage + Canopy light interception at the end of the growth stage) × accumulated incoming radiation during the growth stage × 0.45]. Accumulated incoming radiation was the total sum of the radiation amounts received each day within a specific time period. RUE was defined as the ratio of the accumulated aboveground dry weight during the growth stage to the IPAR. Solar radiation and minimum and maximum temperatures were recorded daily using Vantage Pro2 (Davis Instruments Corp., Hayward, CA, USA).

### 4.4. Quantifying the Contributions of IPAR, RUE, and HI to Grain Yield

Crop yield is calculated by multiplying biomass and harvest index (HI). Biomass production is determined by the product of radiation intercepted by the crop canopy and RUE. By ignoring the role of photosynthesis in root growth, the equation for yield can be simplified to:

Grain yield = IPAR × RUE × HI(1)

To evaluate and compare the relative impacts of IPAR, RUE, and HI on grain yield across varieties in this study, the equation was analyzed using multiple regression with logarithmic transformations:

ln(Grain yield) = a + b ln(IPAR) + c ln(RUE) + d ln(HI)(2)

Here, the coefficients a, b, c, and d for each variety offer insights into the differences in yield. The standard errors associated with these coefficients indicate whether the varied differences in coefficients exist between the varieties.

### 4.5. Statistical Analysis

Three-way analysis of variance (ANOVA) with R 4.3.1 (Analytical Software, Tallahassee, FL, USA) was used to analyze the experiment data. The means were compared using the least significant difference (LSD) test at the 5% probability level. Figures were made using Origin 2024b (OriginLab, Northampton, MA, USA).

## 5. Conclusions

Both ultrasonic treatment and super rice-specific cultivation techniques could effectively increase the yield of super rice in South China by increasing panicle number and grain-filling rate. The important reason for the increase in yield was that the ultrasonic treatment and the super rice-specific cultivation technique could effectively improve the IPAR and RUE during the tillering stage and after the heading stage, indicating that RUE is one of the main factors limiting the yield potential of super rice in South China, and the ultrasonic treatment and the super rice-specific cultivation technique are effective methods to improve RUE. In addition, the super rice-specific cultivation technique reduced the input of soil fertilizer application by 2 times compared with the conventional cultivation technique, which effectively saved time and labor costs. It is worth further studying how to reasonably improve the RUE of high-RUE varieties through other methods.

## Figures and Tables

**Table 1:** Analysis of variance (ANOVA) of the F-values of grain yield (GY), panicle number (PN), spikelets per panicle (SP), grain filling (GF), grain weight (GW), intercepted photosynthetically active radiation (IPAR), aboveground total dry weight (TDW), radiation use efficiency (RUE), and harvest index (HI).

ANOVA	GY	PN	SP	GF	GW	IPAR	TDW	RUE	HI
Year (Y)	71.97 **	127.25 **	ns	133.60 **	17.76 **	3514.81 **	215.62 **	251.23 **	79.20 **
Variety (V)	559.01 **	100.25 **	86.54 **	78.65 **	200.45 **	4.67 *	198.65 **	212.27 **	185.25 **
Treatment (T)	183.12 **	369.87 **	103.20 **	36.35 **	ns	131.89 **	343.44 **	164.72 **	44.05 **
Y × V	ns	12.84 **	8.14 **	19.50 **	18.28 **	ns	5.32 *	4.79 *	ns
Y × T	ns	34.49 **	4.56 **	9.50 **	ns	ns	ns	6.32 **	ns
V × T	ns	11.01 **	ns	3.58 *	ns	4.77 **	ns	3.73 *	ns
Y × V × T	ns	ns	3.26 *	4.40 *	4.01 *	ns	4.37 *	ns	ns

** significant at *p* < 0.01; * significant at *p* < 0.05; ns, non-significant.

**Table 2:** Growth period (GP), intercepted photosynthetically active radiation (IPAR), and radiation use efficiency (RUE) of two rice varieties at each growth stage under different treatments in 2022 and 2023.

Year (Y)	Variety (V)	Treatment (T)	GP (d)	IPAR (MJ m^−2^)				RUE (g MJ^−2^)			
				MT	MT–FL	FL–MA	WGS	MT	MT–FL	FL–MA	WGS
2022	WFY	CK	97	89.53 c	199.69 c	185.48 b	474.70 c	1.46 b	2.29 a	2.72 c	2.30 c
		T1	97	94.82 bc	208.04 b	187.82 b	490.69 b	1.80 b	2.45 a	3.26 b	2.63 b
		T2	97	97.60 ab	214.57 ab	194.29 a	506.46 a	2.51 a	2.61 a	3.31 b	2.86 a
		T3	97	102.57 a	220.25 a	196.98 a	519.80 a	2.80 a	2.46 a	3.71 a	3.00 a
		Mean	97	96.13 A	210.65 A	191.15 A	497.93 A	2.15 A	2.48 B	3.25 A	2.70 AB
	JNSM	CK	101	76.07 b	215.90 b	180.12 c	472.08 b	1.26 b	2.42 ab	2.07 c	2.10 c
		T1	101	81.67 b	222.99 b	182.76 c	487.42 b	1.25 b	2.59 a	2.61 b	2.37 b
		T2	101	93.26 a	239.27 a	185.91 b	518.44 a	2.33 a	2.29 b	2.77 b	2.47 b
		T3	101	98.02 a	246.10 a	188.93 a	533.04 a	2.53 a	2.40 ab	3.19 a	2.70 a
		Mean	101	87.28 A	231.08 A	184.43 A	502.73 A	1.85 A	2.43 B	2.68 A	2.41 B
2023	WFY	CK	98	92.24 d	129.44 d	147.34 c	369.01 d	1.84 b	2.75 b	3.33 b	2.76 b
		T1	98	95.49 c	132.92 c	148.66 c	377.06 c	1.84 b	3.00 b	3.42 b	2.87 b
		T2	98	99.97 b	139.49 b	155.16 b	394.62 b	2.37 a	3.58 a	3.77 ab	3.35 a
		T3	98	103.19 a	142.84 a	158.24 a	404.28 a	2.53 a	3.69 a	4.00 a	3.52 a
		Mean	98	97.73 A	136.15 C	152.35 B	386.25 B	2.13 A	3.28 A	3.63 A	3.13 A
	JNSM	CK	101	79.57 d	129.44 d	132.57 c	364.24 d	1.83 c	2.83 b	2.34 d	2.44 d
		T1	101	84.76 c	132.92 c	134.33 c	375.63 c	1.61 d	3.03 a	2.55 c	2.54 c
		T2	101	94.30 b	139.49 b	139.21 b	403.02 b	2.45 b	2.80 b	3.08 b	2.81 b
		T3	101	98.45 a	142.84 a	142.23 a	415.52 a	3.00 a	2.84 b	3.57 a	3.13 a
		Mean	101	89.30 A	163.23 B	137.08 C	389.58 B	2.20 A	2.85 AB	2.90 A	2.73 AB

Different lowercase letters and uppercase letters indicate statistical differences among treatments at *p* < 0.05 and *p* < 0.01, respectively.

**Table 3:** Regression coefficients, standard error (SE), significance, and adjusted R2 values from multiple-regression analyses for the correlation between the grain yield (GY) of each rice variety and intercepted photosynthetically active radiation (IPAR), radiation use efficiency (RUE), and harvest index (HI).

GY	Intercept		IPAR			RUE			HI		Adj.R2
		Value	SE	*p*-Level	Value	SE	*p*-Level	Value	SE	*p*-Level	
WFY	−4.669	0.798	0.107	**	0.793	0.094	**	0.063	0.185	ns	0.854
JNSM	−4.379	0.985	0.122	**	0.811	0.092	**	−0.234	0.214	ns	0.925
Mean	−5.747	0.439	0.102	**	0.695	0.091	**	0.327	0.136	**	0.868

** significant at *p* < 0.01; ns, non-significant.

**Table 4:** Comparison of different cultivation techniques.

	Fertilizer	Basic Fertilizer N,P_2_O_5_, and K_2_O (kg^−1^)	Tillering Fertilizer N,P_2_O_5_, and K_2_O (kg·ha^−1^)	Panicle Initiation Fertilizer N,P_2_O_5_, and K_2_O (kg·ha^−1^)	Heading Fertilizer N,P_2_O_5_, and K_2_O (kg·ha^−1^)	Foliar Fertilizer at Heading Stage (kg·ha^−1^)	Fertilization Method
Conventional cultivation technique	Urea (46.7%), rapidly available P (15%), and rapidly available K (60%)	72:48:0	36:0:36	63:0:36	9:0:0	-	Broadcast fertilization
Super rice-specific cultivation technique	Special fertilizer for super rice (N:P_2_O_5_:K_2_O = 15:4:6)	135:36:54	45:12:18	-	-	1.5	

## Data Availability

The data sets supporting the results of this article are included within the article.
